# Supplementary material for: Fusarium Species Associated with Maize Leaf Blight in Heilongjiang Province, China
Source: J Fungi (Basel). 2022 Nov 6;8(11):1170. doi: 10.3390/jof8111170 (PMC9698036; doi:10.3390/jof8111170)
Supplement: Supplementary file 1 [file jof-08-01170-s001.zip › jof-1951688-supplementary.pdf]

Fusarium species associated with maize leaf blight in Heilongjiang province, China

Xi Xu[1], Li Zhang[1], Xilang Yang[1], Guijin Shen[1], Shuo Wang[1], Haolin Teng[1], Chunbo Yang[1], Xueyan Liu[1], Xiangjing Wang[1,2], Junwei Zhao[1*], Wensheng Xiang[1,2*]

[1] Key Laboratory of Agricultural Microbiology of Heilongjiang Province, Northeast Agricultural University, No. 600 Changjiang Road, Xiangfang District, Harbin 150030, P.R. China
[2] State Key Laboratory for Biology of Plant Diseases and Insect Pests, Institute of Plant Protection, Chinese Academy of Agricultural Sciences, Beijing 100097, P.R. China
* Email: xiangwensheng@neau.edu.cn; zhaojunwei@neau.edu.cn

Content:

Supplementary Table S1

Supplementary Table S2

Supplementary Table S3

Table S1. *Tef1* gene sequences similarity to reference strain.

Species	*tef1*
F. ipomoeae	98.83-100
F. compactum	99.22-100
F. flagelliforme	96.09
F. citri	99.61
Fusarium sp.	100
F. armeniacum	100
F. sporotrichioides	98.56
F. asiaticum	100
F. graminearum	100
F. acuminatum	100
F. verticillioides	99.60
F. temperatum	100
F. glycines	99.61
F. annulatum	100

Table S2. Disease index and disease incidence on maize leaves inoculated with different *Fusarium* isolates.

Isolates	Species	Disease index	Disease incidence
HA-z142	*F. ipomoeae*	61.67	37.06
HA-z11	*F. ipomoeae*	52.78	24.00
HA-z12	*F. ipomoeae*	58.78	34.17
HA-z13	*F. ipomoeae*	73.78	49.17
HA-z14	*F. ipomoeae*	61.67	37.06
HA-z15	*F. ipomoeae*	62.78	38.17
HA-z16	*F. ipomoeae*	75.28	50.67
HA-z17	*F. ipomoeae*	72.88	48.27
HA-z18	*F. ipomoeae*	64.67	41.06
HA-z19	*F. ipomoeae*	59.78	38.17
HA-z20	*F. ipomoeae*	58.78	36.17
HA-z21	*F. ipomoeae*	75.88	56.10
HA-z22	*F. ipomoeae*	62.07	43.26
HA-x22	*F. ipomoeae*	62.78	38.17
HA-xy82	*F. ipomoeae*	62.88	38.27
HA-xy83	*F. ipomoeae*	74.68	52.07
HA-31	*F. ipomoeae*	63.67	39.06
SH-11	*F. ipomoeae*	58.78	34.17
SH-63	*F. ipomoeae*	60.78	37.17
WC-31	*F. ipomoeae*	77.78	62.50
QQ-41	*F. ipomoeae*	61.67	38.06
SH-62	*F. ipomoeae*	57.68	33.07
HA-z201	*F. ipomoeae*	62.78	38.17
HA-21	*F. ipomoeae*	75.78	51.17
HA-22	*F. ipomoeae*	61.67	37.06
HA-x21	*F. ipomoeae*	59.78	35.17
HA-212	*F. ipomoeae*	52.78	28.17
DQ-n22	*F. ipomoeae*	71.78	47.17
JX-21	*F. ipomoeae*	59.67	35.06
DQ-n31	*F. ipomoeae*	62.78	38.17
HA-61	*F. compactum*	75.36	31.85
HA-111	*F. compactum*	58.12	25.10
JX-y11	*F. compactum*	73.62	46.29
HA-621	*F. compactum*	58.81	32.95
SYS-31	*F. compactum*	54.12	46.26
HA-z152	*F. compactum*	73.25	38.40
HA-z31	*F. compactum*	59.91	41.05
HA-z32	*F. compactum*	73.22	47.16
HA-z33	*F. compactum*	65.36	46.40
HA-z34	*F. compactum*	68.01	38.05
HA-z35	*F. compactum*	74.12	47.16
HA-z36	*F. compactum*	73.36	38.34
HA-z37	*F. compactum*	65.01	36.05
HA-z38	*F. compactum*	74.12	41.16
HA-z39	*F. compactum*	65.30	48.40
HA-z310	*F. compactum*	63.01	34.05
HA-z311	*F. compactum*	68.12	47.16
HA-z312	*F. compactum*	75.36	46.40
HA-xy151	*F. compactum*	61.01	42.95
HA-xy31	*F. compactum*	74.12	47.16
HA-a11	*F. compactum*	73.36	46.40
HA-42	*F. compactum*	69.91	36.05
JX-52	*F. compactum*	74.12	31.16
JX-121	*F. compactum*	73.36	63.50
JX-31	*F. compactum*	63.01	46.66
HA-x12	*F. citri*	55.63	24.00
QTH-21	*F. citri*	77.78	65.20
HA-z1125	*F. citri*	59.75	36.43
HA-z171	*F. citri*	73.95	50.63
HA-z172	*F. citri*	65.63	41.31
HA-z173	*F. citri*	71.50	48.18

Isolates	Species	Disease index	Disease incidence
HA-z174	*F. citri*	62.89	39.57
HA-z175	*F. citri*	67.78	44.46
HA-z176	*F. citri*	57.45	34.03
HA-z177	*F. citri*	67.78	44.46
HA-z1126	*F. citri*	72.98	48.36
HA-xy141	*F. citri*	66.74	43.42
HA-z203	*F. citri*	56.14	32.82
HA-x11	*F. flagelliforme*	51.85	23.03
HA-x51	*F. flagelliforme*	74.78	59.50
HA-a31	*F. graminearum*	75.35	60.23
HG-11	*F. graminearum*	85.12	75.30
QTH-23	*F. graminearum*	76.21	57.82
SH-x72	*F. graminearum*	84.67	66.28
SYS-y21	*F. graminearum*	75.72	57.33
SYS-21	*F. graminearum*	83.63	65.24
SYS-141	*F. graminearum*	77.11	58.72
SYS-142	*F. graminearum*	82.23	63.84
SYS-143	*F. graminearum*	78.314	59.92
SYS-144	*F. graminearum*	81.03	62.64
SYS-145	*F. graminearum*	79.12	60.73
SYS-146	*F. graminearum*	88.04	69.65
SYS-147	*F. graminearum*	76.44	58.05
HA-a142	*F. graminearum*	80.30	61.91
SYS-x71	*F. asiaticum*	60.42	42.67
SYS-x91	*F. asiaticum*	74.75	57.02
HA-x72	*F. asiaticum*	61.22	43.98
HG-x62	*F. asiaticum*	73.95	57.71
SYS-x62	*F. asiaticum*	62.72	45.48
SYS-x131	*F. asiaticum*	72.45	55.21
SYS-x132	*F. asiaticum*	63.82	46.58
SYS-x133	*F. asiaticum*	71.25	54.01
SYS-x134	*F. asiaticum*	64.62	47.38
SYS-x135	*F. asiaticum*	70.65	53.41
HA-zh142	*F. temperatum*	60.42	52.47
QTH-X332	*F. temperatum*	74.75	63.48
QTH-X331	*F. temperatum*	61.22	53.19
QTH-X33	*F. temperatum*	73.95	62.73
HA-z113	*Fusarium* sp.	70.54	63.77
HA-b113	*Fusarium* sp.	77.22	74.35
HA-Z1131	*Fusarium* sp.	83.9	69.05
SYS-x11	*F. sporotrichioides*	61.11	31.25
SYS-x61	*F. sporotrichioides*	59.33	30.49
SYS-x1	*F. sporotrichioides*	59.11	30.29
SYS-x2	*F. sporotrichioides*	60.78	30.98
HG-12	*F. sporotrichioides*	59.66	30.67
SYS-33	*F. sporotrichioides*	60.11	30.87
SYS-101	*F. sporotrichioides*	60.58	30.92
SYS-102	*F. sporotrichioides*	59.33	30.49
SYS-103	*F. sporotrichioides*	61.00	31.02
SYS-104	*F. sporotrichioides*	61.11	31.25
SYS-105	*F. sporotrichioides*	58.35	30.15
SYS-51	*F. sporotrichioides*	59.00	30.25
HG-y102	*F. sporotrichioides*	58.34	30.15
HG-DBy101	*F. sporotrichioides*	58.33	30
SH-z61	*F. acuminatum*	62.56	44.21
SH-61	*F. acuminatum*	74.72	61.11
SH-41	*F. acuminatum*	63.74	45.36
HA-a72	*F. acuminatum*	73.54	60.56
HA-a161	*F. acuminatum*	64.87	46.83
HA-a162	*F. acuminatum*	72.41	59.87
HA-a163	*F. acuminatum*	65.93	47.32
HA-a164	*F. acuminatum*	71.35	58.67
HA-a1211	*F. armeniacum*	61.11	32.5
HA-13	*F. armeniacum*	72.22	52.5

Isolates	Species	Disease index	Disease incidence
HA-a121	*F. armeniacum*	61.56	32.5
HA-a122	*F. armeniacum*	64.44	48.5
HL-42	*F. verticillioides*	62.22	33.42
DQ-n32	*F. verticillioides*	73.16	53.2
SH-n12	*F. verticillioides*	62.34	33.57
JX-123	*F. verticillioides*	68.34	50.16
SH-n11	*F. verticillioides*	63.52	36.98
SH-n201	*F. verticillioides*	63.78	37.23
SH-n202	*F. verticillioides*	63.87	37.32
SH-n203	*F. verticillioides*	70.16	48.32
SH-n204	*F. verticillioides*	65.22	39.62
SH-n205	*F. verticillioides*	63.16	35.87
JX-3352	*F. glycines*	67.78	45.05
JX-335	*F. glycines*	68.87	45.19
HA-171	*F. glycines*	71.32	45.83
HA-172	*F. glycines*	72.11	46
HA-173	*F. glycines*	61.11	44
HA-174	*F. glycines*	70.35	45.62
WC-b53	*F. glycines*	65.83	44.49
HA-z1412	*F. glycines*	64.90	44.22
WC-22	*F. annulatum*	63.93	40.50

Table S3. DNA polymorphism data for FIESC isolates based on *tef1* gene sequences.

**Polymorphism parameter**	***tef1***
Number of isolates	70
Analyzed region	1-263
Number of haplotypes, H	7
Number of polymorphic sites, S	28
Number of mutations, Eta	28
Singleton variable sites	0
Haplotype diversity, *h*	0.759
Nucleotide diversity, π	0.02396
Average number of nucleotide differences, *k*	5.966
Tajima's D	0.08450 ($p > 0.10$)
Fu and Li's D	1.86276 ($p < 0.02$)
Fu and Li's F	1.43557 ($p > 0.10$)
